# Management strategy for hematological malignancy patients with acute respiratory failure

**DOI:** 10.1186/s40001-021-00579-7

**Published:** 2021-09-17

**Authors:** Li Jiang, Qunfang Wan, Hongbing Ma

**Affiliations:** 1 Department of Respiratory and Critical Care Medicine, West China Hospital, Sichuan University, Chengdu, China; 2 Department of Hematology, West China Hospital, Sichuan University, Chengdu, China

**Keywords:** Hematological malignancy, Acute respiratory failure, Intensive care unit, Ventilation

## Abstract

Acute respiratory failure (ARF) is still the major cause of intensive care unit (ICU) admission for hematological malignancy (HM) patients although the advance in hematology and supportive care has greatly improved the prognosis. Clinicians have to make decisions whether the HM patients with ARF should be sent to ICU and which ventilation support should be administered. Based on the reported investigations related to management of HM patients with ARF, we propose a selection procedure to manage this population and recommend hematological ICU as the optimal setting to recuse these patients, where hematologists and intensivists can collaborate closely and improve the outcomes. Moreover, noninvasive ventilation (NIV) still has its own place for selected HM patients with ARF who have mild hypoxemia and reversible causes. It is also crucial to monitor the efficacy of NIV closely and switch to invasive mechanical ventilation at appropriate timing when NIV shows no apparent improvement. Otherwise, early IMV should be initiated to HM with ARF who have moderate and severe hypoxemia, adult respiratory distress syndrome, multiple organ dysfunction, and unstable hemodynamic. More studies are needed to elucidate the predictors of ICU mortality and ventilatory mode for HM patients with ARF.

## Introduction

Hematologic malignancy (HM) is neoplastic myeloid or lymphoid disease, including acute and chronic leukemia, lymphoma, myeloma, as well as myelodysplastic syndrome and myeloproliferative neoplasm [1, 2]. The prognosis of patients with HM has been dramatically improved by chemotherapy and hemopoietic stem cell transplantation [3]. However, therapy-associated pulmonary complications compose 20% undesirable outcomes [3]. Due to pneumonia, sepsis, leukemia infiltration or graft vs. host disease, acute respiratory failure (ARF) is a common pulmonary complication for patients with HM as well as the major reason for intensive care unit (ICU) admission [4, 5]. The mortality varied from 30 to 70% in different reports [4, 6]. Confronting HM patients with ARF in clinical setting, the clinicians have to make decisions about the next procedures. Do they need treatment in an ICU? Which kind of respiratory support should be selected for the patients, noninvasive ventilation (NIV) or invasive mechanical ventilation (IMV)? In this review, based on the results of different investigations related to management of HM patients with ARF, which is defined as PaO_2_ < 60 mmHg, or tachypnea > 30/min, or SpO_2_ < 90% on room air, or the ratio of arterial oxygen partial pressure to fractional inspired oxygen (PO_2_/FiO_2_) < 300, or labored breathing, or respiratory distress, or dyspnea at rest [7–10]we propose a selection strategy to help manage this population and hopefully improve their outcomes.

## Critical care for HM patients with ARF

Undoubtedly, ICU is the best place for critically ill HM patients with ARF, because they can provide high level of life support [3]. As a result, the outcomes in ICU were better than in ward [11, 12]. Respiratory management in ICU was also related to successful extubation for HM with ARF who received mechanical ventilation [13]. In addition, earlier ICU admission (time between ARF onset and ICU admission less than 24 h) leads to better hospital survival [14]. Conversely, delayed admission (more than 2 days) increased the morality of this population [15–17]. However, ICU resource is limited with high cost, HM patients usually spend more critical care resource than non-HM patients [18]. It is also unethical to send end-of-life patients to ICU only for prolonging time. Therefore, it is necessary to screen eligible patients based on reasonable triage policy.

In clinical practice, the determination is mainly made on clinical judgement of physicians. However, some investigators hold that the admission of HM patients to ICU should be determined by objective mortality prediction model rather than clinical experience [18]. The former includes Acute Physiology and Chronic Health Evaluation (APACHE) II scores, the Sequential Organ Failure Assessment (SOFA), and the Simplified Acute Physiology Score (SAPS) II which could predict ICU mortality accurately [19–21]. Namendys-Silva et al. reported the ICU mortality of HM patients who had three or more organ dysfunctions and a SOFA score of 10 points were 70.1% and 80%, respectively. Consequently, HM patients with ARF who had two or fewer organ dysfunctions or a SOFA score of less than 10 points were recommended to ICU admission [6]. However, several studies also demonstrated these scores neither well discriminate the illness severity nor predict the prognosis of this population [22–24]. Azoulay et al. proposed 10 subgroups of HM patients who unlikely benefited from ICU management, but they also emphasized the criteria should not be an obstacle for ICU admission referral, because decision making could not be completely objective; furthermore, new emerging evidence may change our practice [15]. Currently, a feasible way to evaluate the prognosis should be based on combination of clinical experience and matched results of clinical studies as well as the HM patients and the relatives’ willing. Prioritization of ICU admission should be given to those who probably benefit most from critical care. More studies are needed to clarify the value of different score systems in predicting the ICU outcome as well as the criteria of defining early ICU admission for HM patients with ARF.

An ideal ICU for HM with ARF patients should be hematologic ICU, where hematologist, intensivist and respiratory therapist can collaborate closely to provide the optimal critical care [15, 25]. The hematologists are good at addressing HM and related complications, while intensivists and respiratory therapists are accomplished in respiration and circulation support to stabilize patients [15, 18]. They can discuss and make decisions together for HM patients without time and space limitations. However, to our knowledge, most tertiary hospitals in China have general ICUs instead of hematological ICUs. The diagnosis and treatment are split into two parts in two departments, intensivists communicate with hematologist through intermittent and untimely interviews, which can’t guarantee the HM patients at ICU receive the same level of hematological expertise. In fact, many newly diagnosed patients even with life-threatening HM-associated complications had a high survival when they initiate chemotherapy in the ICU [26]. Consequently, when hematological ICU is not available, it plays a crucial role in improving the survival of HM patients with ARF to establish effective and sustained collaboration between hematologist and intensivist.

## Ventilation mode for HM with ARF

Oxygen alone, NIV and IMV compose the common types of respiratory ventilation for HM with ARF. Due to the immune deficiency, HM patients with ARF had a high mortality of 50–70% when they received IMV [27, 28]; therefore, avoiding IMV is a critical strategy to improve the prognosis of this population. NIV, including continuous positive airway pressure (CPAP) and bi-level positive airway pressure (BiPAP), has been recommended for Chronic obstructive pulmonary disease exacerbation, cardiac pulmonary edema and immunosuppressed patients, which can reduce the need of intubation and related complications [27, 29–31]. Usually, BiPAP is better for patients with type II respiratory failure [4].

## NIV for HM with ARF

Several studies have demonstrated NIV can benefit HM patients with ARF (Table 1) [3, 32–40]. Conti et al. evaluated the efficacy of BiPAP among 16 HM patients with ARF (87 ± 22 of arterial partial pressure of oxygen to fraction of inspired oxygen, (PaO_2_/FiO_2_)) in a pilot study. 15/16 patients showed improvement in blood gas and respiration. 5 died and 11 were discharged in good condition. It indicated NIV was a feasible alternative of IMV for HM patients with ARF [34]. Gristina et al. analyzed retrospectively 1302 patients with HM and ARF in 158 Italian ICUs. 21% patients received NIV and more than half (54%) avoided intubation. The mortality in NIV group was significantly lower than immediate IMV and IMV after NIV failure (42% vs. 69% and 77%, respectively). Delayed IMV was related to a little higher mortality than immediate IMV but without significant difference. Based on these facts, they suggested NIV as first line for HM with ARF [32]. Multivariate analysis indicated illness severity and acute lung injury / ARDS at admission were risk factors of NIV failure [32]. Belenguer et al. [36] analyzed retrospectively 41 HM with ARF and compared the outcome of IMV (35 patients) and NIV(6 patients) group in ICU. The mortality was 100% and 37% in the IMV and NPPV group, respectively. Similar outcomes were verified in children HM with ARF and other studies [33]. Therefore, NIV substantially decreases the intubation rate and mortality rate of HM patients with ARF.

**Table 1:** Studies that support NIV in HM patients with ARF

Study	Design	Patients	Setting	Inclusion criteria	comparison	Rate of NIV failure	HRs of NIV failure	Mortality	Comments
Conti, 1998	Prospective	16	ICU	PO_2_ ≤ 60 with FiO_2_ ≥ 0.5, RR ≥ 35	N/A	6.6% (1/16)	N/A	31.3% (5/16)	NIV reduced need of IMV
Hilbert, 2001	RCT	52(30 HM)	ICU	lung infiltration, fever, PO_2_/FiO_2_ < 200, RR > 30	Oxygen/NIV	46%	Severe acidosis, encephalopathy, hemodynamic instability, copious secretions	NIV: 38%, Oxygen: 69%	Early NIV decreased need of intubation and mortality
Piastra, 2004	Prospective	4	ICU	PO_2_/FiO_2_ < 200, RR > 30	N/A	0	N/A	50% (2/4)	NIV decreased need of IMV in pediatric HM patients
Squadrone, 2010	RCT	40	Ward	lung infiltration, SaO_2_ < 90% and RR > 25	Oxygen/NIV	0	N/A	Oxygen: 75%, NIV: 15%	Early NIV reduced need of intubation and ICU admission
Gristina, 2011	Retrospective	1302	ICU	HM with ARF	NIV/IMV	46%	illness severity, acute lung injury / ARDS at admission	NIV success:42%, NIV failure: 77%, IMV: 69%	Recommend NIV as first-line for HM with ARF
Molina, 2012	Prospective	300	ICU	HM patients who needed ventilation support	N/A	60.30%	younger, non-congestive heart failure, bacteremia	NIV: 42.3%; IMV: 72.2%; NIV failure: 79.7%	NIV was preferred for ARF with reversible causes
Belenguer, 2013	Retrospective	41	ICU	HM patients who needed ventilation support	NIV/IMV	40%	N/A	NIV: 37%; IMV: 100%	NIV decreased mortality compared with IMV
Rathi, 2017	Retrospective	1614(899HM)	ICU	PO_2_/FiO_2_ < 200	N/A	38%	younger, high SOFA, HM, BiPAP, non-Caucasian race	NIV failure: ICU mortality:71.3%, hospital mortality: 79.5%	NIV success was associated with the best outcomes; early or late intubation had the same outcomes
Barreto, 2020	Prospective	82	ICU	PO_2_/FiO_2_ < 300, RR > 32	Oxygen/NIV/IMV	50.80%	high SOFA and RR, sepsis	NIV:49.2%; IMV:83.3%; Oxygen: 5.9%	NIV was feasible for HM patients though benefit was controversial

*PaO*_*2*_ arterial oxygen tension, *FiO*_*2*_ fraction of inspired oxygen, *RR* respiratory rate, *IMV* invasive mechanical ventilation, *NIV *noninvasive ventilation, *RCT* randomized control trial, *HM* hematological malignancy, *ARF* acute respiratory failure, *SOFA* sequential organ assessment, *N/A* not available, *BiPAP* bi-level positive airway pressure

However, inconsistent data has also showed NIV may not protect HM with ARF (Table 2) [1, 7, 8, 41–44]. In a retrospective study, Depuydt et al. enrolled 166 HM patients with ARF who required MV in ICU. Based on an analysis of NPPV and IMV with a ratio of 1:2, no difference in mortality was found between 2 groups [44]. However, PO_2_/FiO_2_ in NIV was lower than IMV (72 vs. 147, respectively), indicating NIV group had a high risk of intubation and mortality. Therefore, the baselines in two groups are not comparative. In 2010, the same team compared the efficacy of NPPV, IMV and oxygen alone among 137 HM with ARF. The ICU mortality was 71%, 63%, and 32% as well as in-hospital mortality was 75%, 80%, and 47% in NPPV, IMV and oxygen alone, respectively (*P* = 0.001). The outcomes were determined by the severity of disease rather than the respiratory support type. NIPPV seems to be unprotective to HM with ARF [43]. In a randomized trial, Lemiale et al. enrolled 374 immunosuppressed patients (including 283 HM) with ARF (PO_2_/FiO_2_ 200–300) and compared NIV with oxygen therapy. Compared with oxygen therapy, early NIV did not reduce the 28-day mortality either overall or in subgroups (24.1% and 27.3% in NIV and oxygen, respectively) and subsequent intubation (38.2% and 44.8% in NIV and oxygen, respectively). The authors also acknowledged the lower mortality in oxygen group than expected may limit the power to draw a significant difference in mortality, and the respiratory condition was less severe considering the RR of 25–27 breaths/min [8]. When only 380 HM patients were analyzed prospectively, they still found no benefit could be achieved from NIV compared to oxygen alone [7]. Moreover, many studies revealed a high NIV failure rate of 54–75% in HM with ARF [1, 41, 45], those who failed NIV had a similar or even worse prognosis in patients who initially received IMV [32, 37, 39, 46].

**Table 2:** Studies that do not support NIV in HM patients for ARF

Study	Design	Patients	Setting	Inclusion criteria	comparison	Rate of NIV failure	HRs of NIV failure	Mortality	Comments
Depuydt, 2004	Retrospective	166	ICU	HM patients who needed ventilation support	NIV/IMV	69%	N/A	NIV:65.4%, NIV failure:91.7% IMV: 65.4%	IMV should considered for HM with ARF, especially when ICU admission was driven by bacteremia
Adda, 2008	Retrospective	99	ICU	PO_2_/FiO_2_ < 300	N/A	54%	high RR under NIV, longer delay between admission and NIV, need for vasopressors or RRT, and ARDS	NIV success: 41%; NIV failure: 79%	NIV failure was associated with increased mortality and complications. Predictors of NIV failure can be used to guide intubation
Depuydt, 2010	Retrospective	137	ICU	PO_2_/FiO_2_ < 200	Oxygen/NIV/IMV	75%	N/A	ICU mortality: NIV:71%, IMV:63%, Oxygen:32%	Mortality was determined by severity of illness rather than initial ventilation support
Wermke, 2012	RCT	86(allo-HSCT)	wards	PO_2_/FiO_2_ < 300, SO_2_ < 92%, RR > 25	Oxygen/NIV	76%	N/A	100-day mortality: NIV: 39%; Oxygen: 32%	NIV did not reduce need of intubation and mortality, but study design limited the efficacy
Lemial, 2015	RCT	374(283 HM)	ICU	Immunocompromised patients with ARF	Oxygen/NIV	38.20%	N/A	28-day mortality: NIV: 24.1%; Oxygen:27.3%	Early NIV couldn't reduce 28-day mortality compared to oxygen alone
Lemial, 2015	Prospective	380	ICU	SO_2_ < 90%, or RR > 30	Oxygen/NIV	29%	N/A	NIV: 27%; Oxygen: 25%	NIV did not show benefit for HM with ARF, IMV should not be delayed
Liu, 2017	Retrospective	79	ICU	HM patients who received NIV ventilation	N/A	65%	high FiO_2_, high PCO_2_, vasopressor use	NIV success:21%; NIV failure: 74%	NIV failure was associated with high mortality

*PaO*_*2*_ arterial oxygen tension, *FiO*_*2*_ fraction of inspired oxygen, *RR* respiratory rate, *IMV* invasive mechanical ventilation, *NIV* noninvasive ventilation, *RCT* randomized control trial, *HM* hematological malignancy, *ARF* acute respiratory failure, *N/A* not available, *PCO*_*2*_ partial pressure of carbon dioxide

## IMV for HM with ARF

Although IMV is associated with high mortality of HM patients in ICU [2, 13, 28, 42, 47–49], some studies also indicated early IMV may decrease mortality [37, 44, 50]. Molina et al. enrolled 300 HM with ARF in a prospective, multicenter study. The patients who received IMV after INV failure had a higher mortality than those who received IMV initially [37, 51]. In a randomized trial, Wermke et al. recruited 86 allogeneic hematopoietic stem cell transplantation (allo-HSCT) patients with ARF and compared NIV with oxygen alone, NIV did not reduce the need of intubation and admission to ICU as well as mortality. All intubated patients after NIV failure died. A limitation is that 16/17 patients failing on oxygen alone were switched to NIV, which may attenuate the effect of NIV [42]. However, it can be inferred from the studies early IMV may benefit some patients. In addition, it was reported IMV within 24 h of ICU admission was associated with a better outcome [44, 50]. Most importantly, the critical care level and IMV therapy have been improved tremendously in the past 2 decades and developed continuously, as revealed by the huge reduction of the mortality in ICU and hospital [52]. Many classic predictors, such as neutropenia, APACHE II score, age, and allo-HSCT, have lost their predictive value for HM in ICU [15, 48, 53]. Therefore, early IMV should be the first-line option for this population who have high risk of NIV failure.

Several risk factors that could predict NIV failure have been identified. Barreto et al. [35] implemented IMV, NIV and oxygen only to 82 HM with ARF based on PaO2/FiO2 and clinical judgement, 59 (72%) patients received NIV, and 30 of them (58.2%) need intubation. The mortality was 83.3%, 49.2% and 5.9% in IMV, NIV and oxygen only, respectively. NIV failure was associated with high SOFA(> 7 points), RR (> 34 breaths/min) and sepsis. IMV is recommended if one of these factors exists. Depuydt et al. appealed to IMV for HM with ARF who were excluded from NIV, especially for those whose admission to ICU was driven by sepsis [44]. Other risk factors of NIV failure were also reported in different investigations, which included need for vasopressor, longer delay between admission and NIV, acute respiratory distress syndrome (ARDS), hepatic failure, hemodialysis, high APACHE II and SAPS II score, et al. [4, 35, 37, 39, 41, 45, 54–56].

## The selection of ventilation mode for HM with ARF

Overall, Conflicting conclusions derive from the heterogeneity of different studies. The design, NIV timing and setting, ICU admission policy, inclusion criteria, etiology and organ failure varied among the investigations [5, 54]. Azoulay et al. [57] believed, due to the increasing progress in IMV and remarkable reduced mortality of IMV, it is not necessary to implement NIV for HM patients with ARF. However, significant difference in mortality is still evident between NIV and intubation either in 2000 (50% vs. 90%) or in 2010(15% vs. 60%) although mortality rate decreases over time [57]. Based on the available data and our experience, NIV still has its own place for HM with ARF when it is initiated earlier and used for selected patients.

Squadrone et al. reported early use of CPAP in the ward can significantly reduce the need of ICU admission and subsequent intubation. 40 HM patients with neutropenia and mild respiratory failure (200–300 of PaO_2_/FiO_2_) were enrolled and assigned randomly to oxygen and CPAP group. The inclusion criteria included radiological evidence of bilateral pulmonary infiltration due to non-infectious causes, SaO2 < 90% in room air, and respiratory rate > 25 breaths/min, mortality rate was 75% and 15% in oxygen and CPAP, respectively [3]. Another randomized trial reported by Hilbert et al. also showed similar results in 52 immunosuppressed patients (including 30 HM) [38]. These results illustrate HM patients can benefit from NIV when ARF is mild, NIV may lost its potential value when ARF is severe [44]. In addition, selecting eligible HM patients plays critical role in increasing NIV success rate. As mentioned above, the patients who have no or few high risk of NIV failure may benefit most from the NIV.

**Fig. 1:**
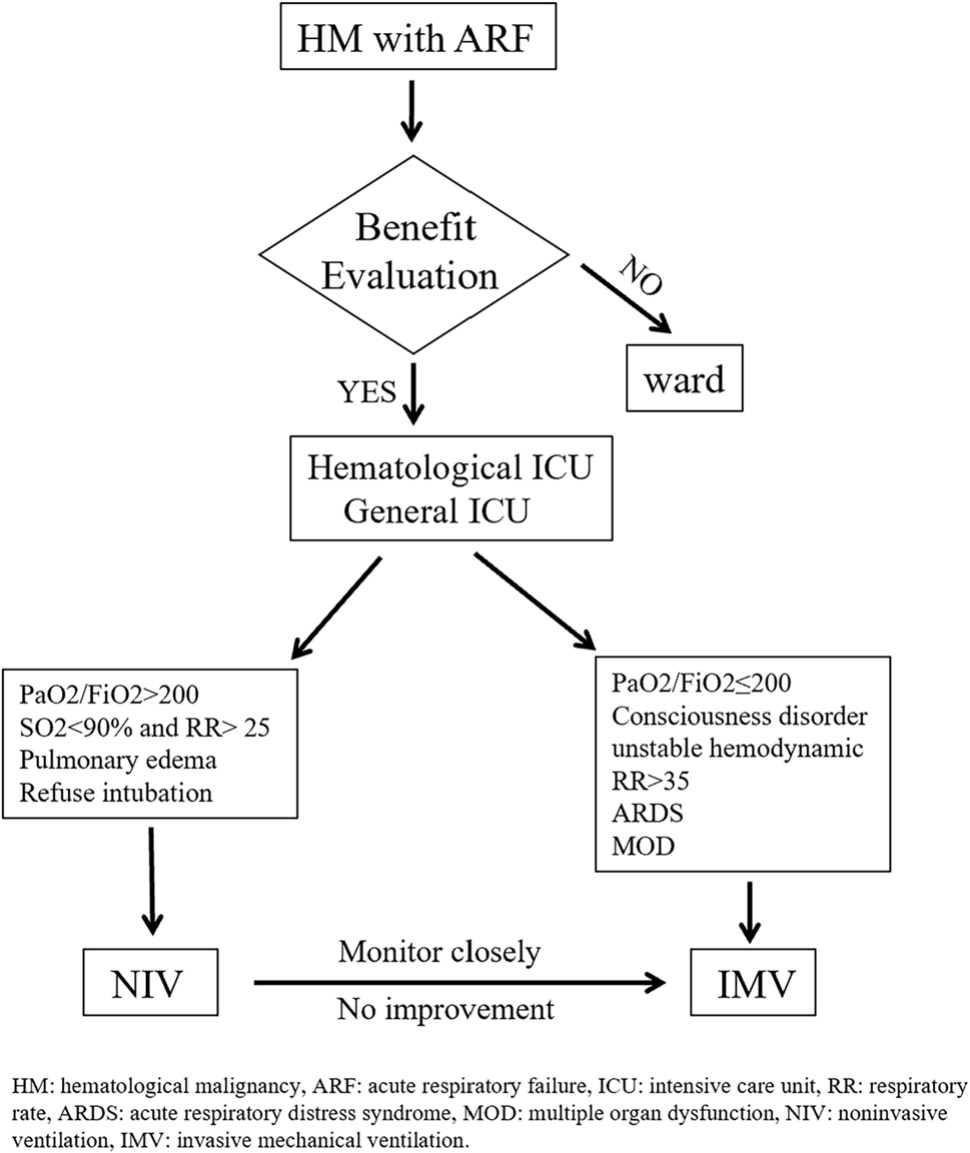
Suggested flow for HM patients with ARF

Although the predictive indicators are not completely consistent and need more high-quality trials to prove, a comprehensive suggestion can be outlined. NIV is preferred for HM patients with PaO_2_/FiO_2_ > 200 or SO2 < 90% and RR > 25 breaths/min, and those who have reversible etiology, such as cardiac pulmonary edema or refuse intubation [4, 37, 38, 54] (Fig. 1). In addition, careful adjustment of NIV administration in the first hours to improve patient tolerance and avoid leaks could result in better outcome [25], which emphasize the experience and organization of team group [4]. Usually, alleviation in dyspnea and improvement in artery blood gas analysis could be achieved within 2 h after NIV implementation if it was effective [58], improved PaO_2_/FiO_2_ after 1 h was a predictor of NIV success [56]. Consequently, close evaluation of NIV efficacy and early switch to IMV are critical when NIV is properly administered and shows no improvement (Fig. 1). In contrast, IMV should be the first option for those consciousness disorder, unstable hemodynamic, PaO_2_/FiO_2_ < 200, RR > 35 breaths/min, ARDS and multiple organ dysfunction (Fig. 1) [4, 35, 37, 39, 41, 45, 54, 57].

## Conclusions

HM patients with ARF have a high mortality rate. Hematological ICU is the optimal place to rescue this patient population. Admission to ICU should be as early as possible when they may benefit from critical care by evaluation. Close collaboration among hematologists, intensivist, respiratory therapists and other physicians plays a pivotal role in providing high level of diagnosis and treatment and in producing better outcomes. NIV still has its own place for selected HM patients with ARF who have mild hypoxemia and reversible causes. It is also crucial to monitor closely the efficacy of NIV and switch to IMV at appropriate timing when NIV shows no apparent improvement. Otherwise, early IMV should be initiated to HM with ARF who have moderate and severe hypoxemia, ARDS, MOD, and unstable hemodynamic (Table 3). More studies are needed to elucidate the predictors of ICU mortality and ventilatory mode for HM patients with ARF.

**Table 3:** Recommendations for HM patients with ARF

ICU admission	Giving priority to those who may benefit most from critical care based on an integration of clinical experience, matched results of clinical studies and the willing of patients and their relatives. More studies are needed to verify predictors, such as SOFA, APACHEII, SAPSII and others
Optimal setting	Hematological ICU is preferred, or General ICU where hematologist, intensivist and respiratory therapist can collaborate closely
NIV	PaO2/FiO2 > 200; SO2 < 90% and RR > 25; Pulmonary edema; Refuse intubation
IMV	PaO2/FiO2 ≤ 200; RR > 35; Consciousness disorder; unstable hemodynamic; ARDS; MOD

*ICU* intensive care unit, *SOFA* sequential organ failure assessment, *APACHE* acute physiology and chronic health evaluation, *SAPS* simplified acute physiology Score, *NIV* noninvasive ventilation, *PaO*_*2*_ arterial oxygen tension, *FiO*_*2*_ fraction of inspired oxygen, *SO2* oxygen saturation, *RR* respiratory rate, *IMV* invasive mechanical ventilation, *ARDS* acute respiratory distress syndrome, *MOD* multiple organ dysfunction

## Data Availability

Not applicable.
